# Manual therapy for idiopathic scoliosis: A protocol for systematic review and meta-analysis

**DOI:** 10.1097/MD.0000000000021782

**Published:** 2020-08-21

**Authors:** Qian Huang, Lei Zhang, Zhiwei Li, Lingjun Kong

**Affiliations:** a Department of Acupunctue and Tuina, Lianyungang TCM Hospital Affiliated to Nanjing University of Chinese Medicine, Lianyungang; b Yueyang Hospital of Integrated Traditional Chinese and Western Medicine, Shanghai University of Traditional Chinese Medicine, Shanghai, China.

**Keywords:** manual therapy, idiopathic scoliosis, meta-analysis, systematic review

## Abstract

**Introduction:**

More patients with idiopathic scoliosis (IS) preferred to choose manual therapy as a complementary conservative treatment, but the effects of manual therapy for IS remains controversial. The previous reviews could not draw reliable conclusion due to few eligible studies to perform a meta-analysis. In the last decade, however, several new studies were published that assessed the effects of manual therapy in the management of IS, especially in China. Therefore, the present systematic review and meta-analysis will be performed to examine whether manual therapy is effective for IS primarily in improving patient-centerd symptoms and secondarily in radiographic outcomes.

**Methods and analysis:**

A computerized literature search will be performed in the following electronic databases from their inceptions to June 2020 to identify randomized controlled trials of manual therapy in the management of IS: PubMed, EMBASE, MEDLINE, Cochrane Central Register of Controlled Clinical Trials, China Knowledge Resource Integrated Database, Wanfang Data Information, and Weipu Database for Chinese Technical Periodicals. The quality of included studies will be assessed independently by 2 reviewers using the Physiotherapy Evidence Database scale. The meta-analysis will be performed with the Review Manager Version 5.3 software to assess the effects on patient-centred outcomes and radiographic outcomes of manual therapy for IS. The heterogeneity will be assessed using *I*^2^ statistic and Cochran *Q* statistic. The subgroup analysis will be conducted based on different control interventions and subpopulations. Quality of evidence will be assessed using the Grades of Recommendation, Assessment, Development and Evaluation.

**Ethics and dissemination:**

No ethical statement will be required for the performance of this review and meta-analysis. The results of this review will be published in an international peer-reviewed journal.

INPLASY registration number: INPLASY202070058

Strengths and limitations of this study

1. To our knowledge, this is the first systematic review including quantitative data synthesis of patient-centred outcomes and radiographic outcomes of the patients with idiopathic scoliosis (IS) who are receiving manual therapy.
2. This systematic review will only include the evidence from randomized controlled trials of manual therapy for IS in order to examine whether manual therapy is an effective conservative intervention in the management of IS. Most of previous reviews, however, were based on observational case reports and small-scale clinical studies.
3. Subgroup analyses may not be possible due to a lack of data (less than three studies). This may limit the recommendations of manual therapy in the management of IS based on different control interventions (such as bracing or exercise) and subpopulations (adults or adolescent).

## 1 Introduction

Idiopathic scoliosis (IS) is a common spinal disorder of unknown cause at least a 10° lateral curve of the spine that comprises approximately 80% of diagnosed scoliosis cases.^[1]^ Based on the literatures, the common prevalence of IS was about 2% to 3% worldwide, but there is obviously difference among counties.^[2]^ In Singapore, the overall prevalence rate was only 0.93% in girls and 0.25% in boys.^[3]^ In Brazil, the prevalence of adolescent IS in the public schools of Goiânia was up to 4.3%.^[4]^ In China, however, the prevalence was as high as 5.4% to 6.5% based on more than 700 thousand adolescent students. This spinal deformity could lead to a large number of accompanying comorbidities including kyphosis due to prominent posterior rib angles, back pain, respiratory function impairments, depression, anxiety, and poor quality of life.^[1–6]^ Therefore, the IS patients and primary caregivers usually suffer from considerable distress and burden due to multiple medical and surgical interventions to address the spinal deformity and its accompanying comorbidities.^[7]^

Treatment options for IS usually are surgery or conservative treatments including observation, spinal orthosis, exercise, and so on.^[8]^ The adolescent IS patients were supported to employ the bracing and physiotherapeutic scoliosis-specific exercises to prevent the progression of scoliosis and improving relevant accompanying comorbidities by 2016 guidelines of the international Society on Scoliosis Orthopedic and Rehabilitation Treatment (SOSORT).^[2]^ The general goals, starting from the most important, of conservative treatments are to improve esthetics, quality of life, disability, back pain, psychological well-being, progression in adulthood, breathing function, scoliosis cobb degrees, and so on. according to SOSORT consensus paper.^[9]^ There was no recommendation on manual therapy for IS in 2016 SOSORT guidelines of orthopedic and rehabilitation treatment of IS during growth, but more and more adolescent IS sufferers preferred to choose manual therapy as a complementary conservative treatment considering the risk of surgery, poor quality of life resulting from the bracing, and uncertainty curve progression risk during observation period.^[10–12]^

Manual therapy is a skilled hand manipulation, including massage, chiropractic, osteopathy, and so on, intended to increase range of motion of the joint, modulate tissue/muscle extensibility, improve soft tissue movement restriction, relieve pain, and promote psychological well-being.^[13]^ Therefore, most patients with IS preferred to choose manual therapy aimed at relieving pain, alleviating negative emotions, treating circulatory and respiratory dysfunctions, improving aesthetics, and then halting curve progression. Several studies reported that manual therapy might contribute to improve pain, abnormal posture, dysfunction, and quality of life in patients with IS.^[14,15]^ However, the effects of manual therapy for IS remains controversial. The previous reviews did not draw reliable conclusion on the effects of manual therapy in the management of IS.^[16–19]^ The effects of manual therapy in the management of IS cannot be reliably evaluated because there were no enough eligible studies to conduct data synthesis in the meta-analysis. Most relevant studies were observational case studies or small-scale clinical trials with poor methodology.^[18]^ With more patients and physicians using manual therapy for IS, however, several randomized controlled trials (RCT) were published that assessed the effects of manual therapy in the management of IS, especially in China.^[20–23]^ Tuina, a traditional Chinese manual therapy, is comprised of soft-tissue manipulations (similar to massage) and adjusting manipulations (similar to chiropractic), that is widely used by practitioners to prevent and treat IS.

The aim of this systematic review is to evaluate the effects of manual therapy in the management of IS. Based on the recommendation of the SOSORT and Scoliosis Research Society (SRS), the clinical study of the conservative therapy for IS should focus primarily on patient-centred outcomes (such as aesthetics, pain, disability, quality of life, etc), and secondarily on radiographic outcomes (Cobb angle, vertebral rotation, etc.).^[24]^ Therefore, the meta-analysis will be conducted to examine the evidence of the effect of manual therapy for IS primarily in improving patient-centred symptoms and secondarily in radiographic outcomes.

## 2 Methods

This review protocol is prepared according to the Preferred Reporting Items for Systematic Reviews and Meta-Analysis guidelines for reporting systematic review and meta-analyses of RCTs. The review was registered on the international platform of registered systematic review and meta-analysis protocols with the registration number international platform of registered systematic review and meta-analysis protocols 202070058. No ethical statement will be required for the performance of this review and meta-analysis. The results of this review will be published in an international peer-reviewed journal.

### 2.1 Search strategy

A computerized literature search will be performed in the following electronic databases from their inceptions to June 2020: PubMed, EMBASE, MEDLINE, Cochrane Central Register of Controlled Clinical Trials, China Knowledge Resource Integrated Database, Wanfang Data Information, and Weipu Database for Chinese Technical Periodicals. The following key terms will be used in combination to develop search strategy for each electronic database: (scoliosis OR spinal curve) AND (manual therapy OR massage OR chiropractic OR osteopathy OR mobilization OR spinal manipulation OR myofascial release OR Tuina OR Shiatsu). The literature search strategy is summarised for PubMed in Table 1. Manual searching will be conducted at the library of Shanghai university of traditional Chinese medicine. The reviewers will screen the reference lists of eligible studies and relevant reviews to identify additional sources of information. Search results will be compiled using the citation management software EndNote X9.

**Table 1:**
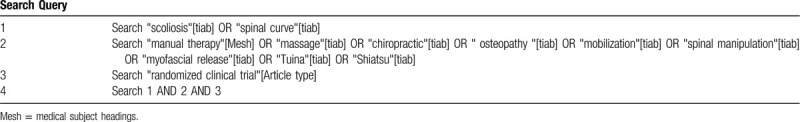
Search strategy (through PubMed).

### 2.2 Selection of eligible studies

**Figure 1:**
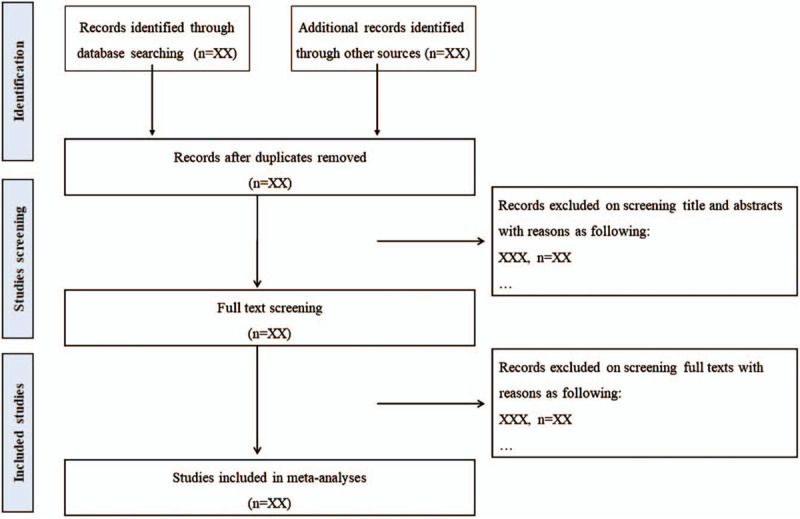
Flow chart of study selection process.

Two reviewers will independently screen the titles and abstracts of the retrieved articles. We will also acquire the full text for screening to evaluate the eligibility for inclusion when necessary. All articles are published in English or Chinese. Any disagreements will be resolved by discussion among reviewers. The process and results of the studies selection will be presented in a flow chart with Figure 1. The inclusion/exclusion criteria are as following:

1. **Type of studies**: the systematic review will include the published RCTs of manual therapy for IS. The crossover design study will also be included in this review, but only the data of the first phase will be included in the meta-analysis. For this review, case reports, observational studies, and cross-sectional design studies of manual therapy for IS will be excluded. The study protocol and conference abstract of RCTs will also be excluded, if the corresponding author could not provide all research data.
2. **Type of participants**: studies will be eligible if they include participants with a diagnosis of IS, spinal disorder of unknown cause at least a 10° lateral curve of the spine. There were no limitations on age, gender, or nationality of the IS patients. The participants with other types of scoliosis (such as congenital, neuromuscular, etc) will be excluded in this review.
3. **Type of interventions**: for this review, the eligible intervention is manual therapy, is a skilled hand manipulation, including massage, chiropractic, osteopathy, mobilization, spinal manipulation, myofascial release, Tuina, Shiatsu, and so on. The control interventions will include observation, bracing, exercise therapy, medicine, education, and any treatments without manual therapy. The included studies may assess the effects of manual therapy compared with other therapy (such as manual therapy versus bracing). The study valuating the effects of manual therapy plus a conservative intervention compared with the same conservative intervention (such as manual therapy plus bracing versus bracing) will also be included. However, the study assessing the effects of manual therapy plus a conservative intervention compared with another conservative therapy (such as manual therapy plus bracing versus traction) will be excluded in this review.
4. **Type of outcomes**: the SOSORT and SRS recommended that the clinical study of the conservative therapy in the management of IS should focus primarily on patient-centred outcomes (such as aesthetics, pain, disability, quality of life, etc), and secondarily on radiographic outcomes (Cobb angle, vertebral rotation, etc). Therefore, the primary outcomes will be patient-centred symptoms including pain, disability, depression, anxiety, quality of life, aesthetics, and so on. The secondary outcomes will include Cobb angle, vertebral rotation, and so on. The studies will be excluded in this review if the outcome only includes the improvement rate of IS patients without other focusing continuous data.

For the eligible studies, the patient-centred outcomes should be assessed by the following standardized scales: assessing pain using Visual Analogue Scale, Brief Pain Inventory, Numerical Rating Scale, and so on, disability using Oswestry Disability Index, Neck Disability Index, etc., depression using Self-rating Depression Scale, Center of Epidemiologic Studies-Depression Scale, and so on, anxiety using Self-rating Anxiety Scale, State Anxiety Inventory, and so on, and quality of life using Short Form-36 Questionnaire, and so on. The comprehensive assessing scale of scoliosis (such as Scoliosis Research Society-22) also is eligible.

### 2.3 Data extraction

Data will be extracted independently by 2 reviewers according to pre-defined criteria. Any discrepancies will be resolved through discussion among reviewers. The following information was extracted:

(1) Study characteristics: first author, year, country, and follow-up time.
(2) Participants information: sample size, mean age, Cobb degrees, and main curve location.
(3) Interventions and comparators: type of manual therapy, control interventions, time of each intervention, intervention sessions, and total treatment duration.
(4) Outcomes: assessing scales of the patient-centred symptoms including pain, disability, depression, anxiety, aesthetics, quality of life, etc. and radiographic outcomes including Cobb angle, vertebral rotation, and so on.

### 2.4 Quality assessment

The quality of the included studies will be assessed independently by 2 reviewers using the Physiotherapy Evidence Database (PEDro) scale. The PEDro scale is a tool developed to measure the methodological quality of RCTs of physiotherapy interventions. The study reported that there was evidence of convergent and construct validity of PEDro scale for physiotherapy trials.^[25,26]^ Reporting of the following aspects will be assessed:

(1) study eligibility criteria specified,
(2) random allocation of subjects,
(3) concealed allocation,
(4) measure of similarity between groups at baseline,
(5) subject blinding,
(6) therapist blinding,
(7) assessor blinding,
(8) less than 15% dropouts,
(9) intention- to-treat analysis,
(10) between-group statistical comparisons, and
(11) point measures and variability data.

The PEDro score will be calculated by criteria (2) to (11) according to meeting the criteria or not, and a cut point of 6 using the PEDro scale indicates high-quality studies. The overall quality of evidence was assessed using the Grades of Recommendation, Assessment, Development and Evaluation framework including the risk of bias, inconsistency, indirectness, imprecision, and publications bias. Any disagreement will be resolved through discussion among reviewers.

### 2.5 Data synthesis and analysis

If possible, the meta-analysis will be performed with the Review Manager Version 5.3 software. For the continuous data, the changes from baseline will be used in the meta-analysis. A random effects model will be used for a better analysis of the clinical heterogeneity. If outcome measure scales are the same, the mean difference and 95% confidence intervals will be calculated. In the case of different outcome measure scales, the standardized mean difference and 95% confidence intervals will be calculated.

According to the recommendations of the Cochrane handbook for systematic reviews of interventions, the heterogeneity will be assessed using *I*^2^ statistic (where *I*^2^ >30% indicated moderate heterogeneity; *I*^2^ >50% substantial heterogeneity; and *I*^2^ > 75% considerable heterogeneity) and Cochran *Q* statistic (considered to be statistically significant when *P*<.10).^[27]^

The subgroup analysis will be conducted based on different control interventions and subpopulations if there are more than 3 eligible studies. The risk of publication bias will be assessed by funnel-plot if more than ten trials are included in the meta-analysis. If relevant data are not reported, the original authors will be contacted to request the missing data, especially for those necessary for the meta-analysis. If the meta-analysis is not possible, a narrative synthesis of the available data will be conducted.

## 3 Discussion

This systematic review will focus on the effects of manual therapy for IS in improving patient-centred symptoms (aesthetics, pain, disability, quality of life, etc), and secondarily in radiographic outcomes (Cobb angle, vertebral rotation, etc) according to the recommendation of the SOSORT and SRS. The previous reviews did not draw reliable conclusion on the effects of manual therapy in the management of IS because it cannot be reliably evaluated the relevant effects due to no enough eligible studies to conduct data synthesis in the meta-analysis.^[16–19]^ In the last decade, however, several new RCTs were published. Therefore, the meta-analysis on the effects of manual therapy for IS could be performed to fill this evidence gap in our review. Therefore, the current study, to our knowledge, may be the first systematic review with data synthesis of RCTs of manual therapy in the management of IS. In this review, manual therapy may be used alone in the management of IS (such as manual therapy for IS), and 1 of the combined conservational interventions for IS (such as manual therapy plus bracing for IS). The relevant effects will be assessed independently if there are enough eligible studies. This systematic review may show the beneficial evidence on the effects of manual therapy, as a complementary conservative intervention, in the management of IS.

## Author contributions

**Conceptualization**: Qian Huang, Lingjun Kong

**Funding acquisition**: Qian Huang, Lingjun Kong

**Investigation**: Qian Huang, Lei Zhang, Zhiwei Li, Lingjun Kong

**Methodology**: Qian Huang, Lei Zhang, Zhiwei Li

**Project administration**: Qian Huang, Zhiwei Li

**Supervision**: Qian Huang, Lingjun Kong

**Writing – original draft**: Qian Huang, Lei Zhang, Zhiwei Li

**Writing – review & editing**: Lingjun Kong
